# Molecular Characterization of the *BMP7* Gene and Its Potential Role in Shell Formation in *Pinctada martensii*

**DOI:** 10.3390/ijms151121215

**Published:** 2014-11-17

**Authors:** Fang Yan, Shaojie Luo, Yu Jiao, Yuewen Deng, Xiaodong Du, Ronglian Huang, Qingheng Wang, Weiyao Chen

**Affiliations:** Fishery College, Guangdong Ocean University, 40 East Jiefang Road, Xiashan District, Zhanjiang 524025, Guangdong, China; E-Mails: yanfanghdxs@163.com (F.Y.); rogerjoke1@hotmail.com (S.L.); gdhddxd@hotmail.com (X.D.); hrl8849@163.com (R.H.); wangqingheng@163.com (Q.W.); A415878754@126.com (W.C.)

**Keywords:** *Pinctada martensii*, bone morphogenetic protein 7 (BMP7), RNA interference, nacre, prismatic layer, shell

## Abstract

Bone morphogenetic protein 7 (BMP7), also called osteogenetic protein-1, can induce bone formation. In this study, the obtained full-length cDNA of BMP7 from *Pinctada martensii* (Pm-BMP7) was 2972 bp, including a 5'-untranslated region (UTR) of 294 bp, an open reading fragment of 1290 bp encoding a 429 amino acid polypeptide and a 3'-UTR of 1388 bp. The deduced protein sequence of Pm-BMP7 contained a signal peptide, a pro-domain and a mature peptide. The mature peptide consisted of 135 amino acids and included a transforming growth factor β family domain with six shared cysteine residues. The protein sequence of Pm-BMP7 showed 66% identity with that from *Crassostrea gigas*. Two unigenes encoding Pm-BMPRI (Pm-BMP receptor I) and Pm-BMPRII were obtained from the transcriptome database of *P. martensii.* Tissue expression analysis demonstrated Pm-BMP7 and Pm-BMPRI were highly expressed in the mantle (shell formation related-tissue), while Pm-BMPRII was highly expressed in the foot. After inhibiting Pm-BMP7 expression using RNA interference (RNAi) technology, Pm-BMP7 mRNA was significantly down-regulated (*p* < 0.05) in the mantle pallium (nacre formation related-tissue) and the mantle edge (prismatic layer formation related-tissue). The microstructure, observed using a scanning electron microscope, indicated a disordered growth status in the nacre and obvious holes in the prismatic layer in the dsRNA-Pm-BMP7 injected-group. These results suggest that Pm-BMP7 plays a crucial role in the nacre and prismatic layer formation process of the shell.

## 1. Introduction

Biomineralization is a widespread process existing in all kingdoms of living organisms. The shell of mollusks is a stable organo-mineral product composed of calcium carbonate and organic matrix, including proteins and polysaccharides [1]. Although the organic matrix, especially proteins, account for less than 5% (*w*/*w*) of the biomineralized shell, they are primarily responsible for controlling the CaCO_3_ polymorph (calcite, aragonite), the size, the shapes of the crystallites, and finally, the texture of the shell [2]. In terms of the shell structure of the bivalve pearl oyster, it includes two mineralized layers: The inner aragonite nacre and the outer calcite prismatic layer, which are mainly regulated by the matrix proteins secreted from the mantle pallium and the mantle edge, respectively. To date, many shell matrix proteins and their corresponding genes have been identified to infer the mechanisms under shell formation [3]. However, shell formation is a very complex, precisive process, and the expression of each related protein is subject to strict regulation. Consequently, the signal molecules and transcription factors involved in shell formation should also be investigated.

Bone morphogenetic proteins (BMPs) are multi-functional growth factors that belong to the transforming growth factor β (TGF-β) superfamily. Since Urist *et al.* discovered BMPs in 1965 as active components that can induce bone formation in demineralized bone extracts [4], over 20 members of BMPs have been found and identified in vertebrates to date. The major function of BMPs is to induce the formation of both bone and cartilage [5,6]. They also play a role in embryogenesis, hematopoiesis and neurogenesis [7]. BMPs have also been identified in invertebrates, such as echinoderms [8,9], arthropods [10,11], mollusks [12,13,14], platyhelminths [15], cnidarians [16,17] and poriferans [18]. Studies have shown that BMPs play similar roles in vertebrates and invertebrates. In the latter, most of the identified BMPs are BMP2/4 and BMP1 homologues. However, limited information about BMP7, one of the main representatives of BMPs with the greatest osteogenic capacity [5], has been reported.

The pearl oyster *Pinctada martensii*, also known as *Pinctada fucata martensii* in Japan, is the main species cultured for marine pearl production in China and Japan. It is a representative research model for biomineralized shell formation. A partial sequence of the *BMP7* gene was obtained in our previous research on the transcriptome of pearl sac from *P. martensii* [19]. The present study was designed to determine the full length of *Pm-BMP7* gene and elucidate its exact functions in shell formation.

## 2. Results

### 2.1. Molecular Cloning and Sequence Analysis of BMP7 from Pinctada martensii (Pm-BMP7)

Based on the 1406 bp fragment from the *P. martensii* transcriptome database, two fragments of 210 and 1527 bp were amplified by the 5'- and 3'-RACE (rapid amplification of cDNA end) using gene-specific primers. A 2972 bp nucleotide sequence representing the complete Pm-BMP7 cDNA, was obtained by overlapping the three fragments above.

**Figure 1:**
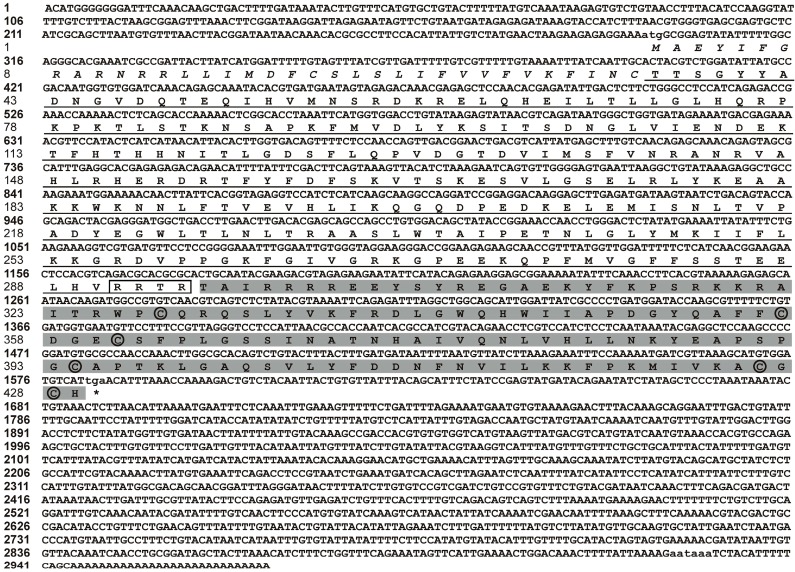
Nucleotide and amino acid sequences of *Pm-BMP7* gene. The bold and normal numbers on the left indicate the positions of the Pm-BMP7 cDNA sequence and the amino acid residues, respectively. The initiation codon (atg), the stop codon (tga), and the putative polyadenylation signal (aataaa) are shown by small letters. The putative signal peptide is indicated in italic, the pro-domain is underlined with boxed putative maturation site, and the mature protein is shaded in gray with circled 6 cysteine residues.

The complete Pm-BMP7 cDNA sequence contained a 5'-UTR of 294 bp, an open reading fragment (ORF) of 1287 bp predicted to encode a 429 amino acid polypeptide, a 3'-UTR of 1391 bp with 28 bp poly (A) tail and a typical polyadenylation signal (aataaa). This cDNA sequence has been submitted to GenBank with the Accession No. AGS32053.1. The analysis of the deduced amino acid sequence showed Pm-BMP7 contained a signal peptide (1–35 aa), a pro-domain (36–294 aa) and a mature peptide (295–429 aa). The mature peptide was produced by cleaving off the pro-domain in the putative maturation site Arg-X-X-Arg [7,20] (Figure 1). The mature protein consisted of 135 amino acids with an estimated molecular mass of 15.60 kDa and a theoretical isoelectric point of 9.51, and included a TGF-β family domain (318–429 aa) with 6 conserved cysteine residues (Figure 1).

### 2.2. Homologous and Structural Analysis of Pm-BMP7

Multiple comparisons using the BLASTx program showed the deduced amino acid sequence from *P. martensii* is homologous to the BMP7 protein of the bone morphogenetic protein family. It shared the highest identity (66%) with BMP7 from *Crassostrea gigas*. Meanwhile, 61% identity was shared with BMP7 from *Tegillarca* *granosa*, 40% from *Xenopus laevis*, 39% from *Danio rerio*, and 39% from *Homo sapiens*.

**Figure 2:**
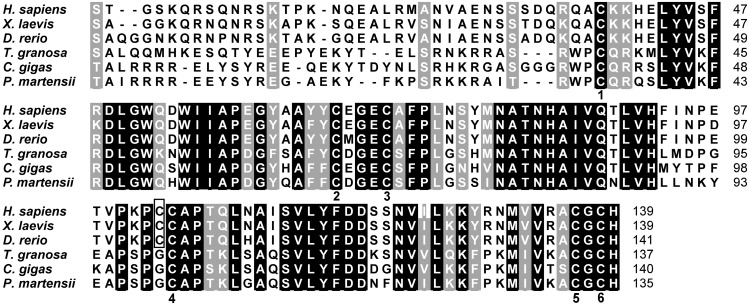
Homology of the Pm-BMP7 between vertebrates and invertebrates. Multiple comparisons of mature BMP7 from different species (accession numbers: NP_001710.1, AAH55959.1, NP_001070614.1, AFP57673.1, EKC34211.1, AGS32053.1). The conserved amino acids in all animal mature BMP7 are written in white on black background, and similar amino acids are shaded in gray. The numbers on the right refer to the total amino acid of each protein. The numbers in the lower part of the alignment sequences indicate the shared cysteine residues between vertebrates and invertebrates. Additional cysteine residues in vertebrates are boxed.

The homologous analysis of mature BMP7 from vertebrates and invertebrates using ClustalX software (Des Higgins and Paul Sharp, Dublin, Ireland) indicated that the carboxyl terminal of Pm-BMP7, namely the TGF-β family domain, shared six cysteine residues, and the spacing between cysteine residues was well conserved among all mature BMP7 (Figure 2).

**Figure 3:**
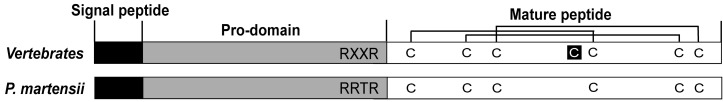
A scheme depicting the structure of Pm-BMP7 protein. It contains signal peptide, a pro-domain and a mature peptide. The vertebrate BMP7 drawing was derived from the analysis of the 49 amino acid sequences (accession numbers: XP_003443653.1, CAN13248.1, NP_001182087.1, XP_004370274.1, XP_004282349.1, NP_001192944.1, XP_001510324.1, XP_001377957.1, ELK04321.1, XP_003787768.1, NP_001183981.1, XP_004585974.1, XP_003419958.1, XP_004717200.1, NP_031583.2, XP_004014818.1, AAH55959.1, CBN81040.1, XP_004636067.1, XP_003501484.1, XP_004840979.1, XP_003463692.1, NP_989197.1, NP_001710.1, XP_002747751.1, XP_004785815.1, XP_004416595.1, XP_004663898.1, EMP35793.1, XP_004430302.1, XP_004081013.1, XP_003932663.1, XP_417496.4, ELK33961.1, NP_001070614.1, XP_003442703.1, XP_004468077.1, XP_004574195.1, XP_003439028.1, XP_003439028.1, XP_003973521.1, XP_003223787.1, XP_004546243.1, AAF34179.1, NP_001029110.1, XP_004070841.1, XP_003963437.1, XP_004881107.1, CBH32479.2). Cysteine residues in black letters are conserved in all BMP7 proteins. The lines above the mature peptide drawing represent the disulfide bonding pattern of BMP7 proteins.

A structure scheme of Pm-BMP7 was drawn according to homology. The six common cysteines between Pm-BMP7 and vertebrates were used to build a cystine knot by forming three intrachain disulfide bonds, whereas the additional cysteine in vertebrates could form an interchain disulfide bond (Figure 3).

### 2.3. Expression Analysis of Pm-BMP7 and Its Receptors

**Figure 4:**
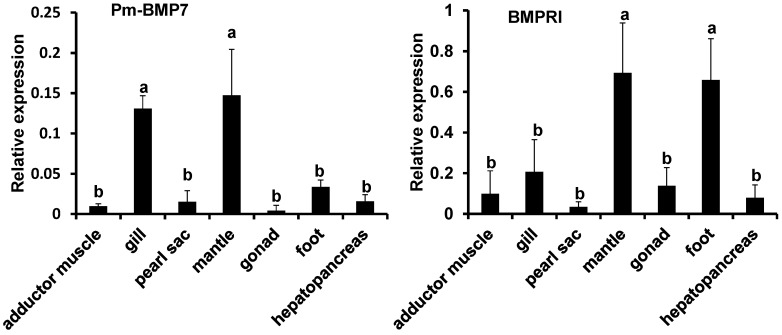

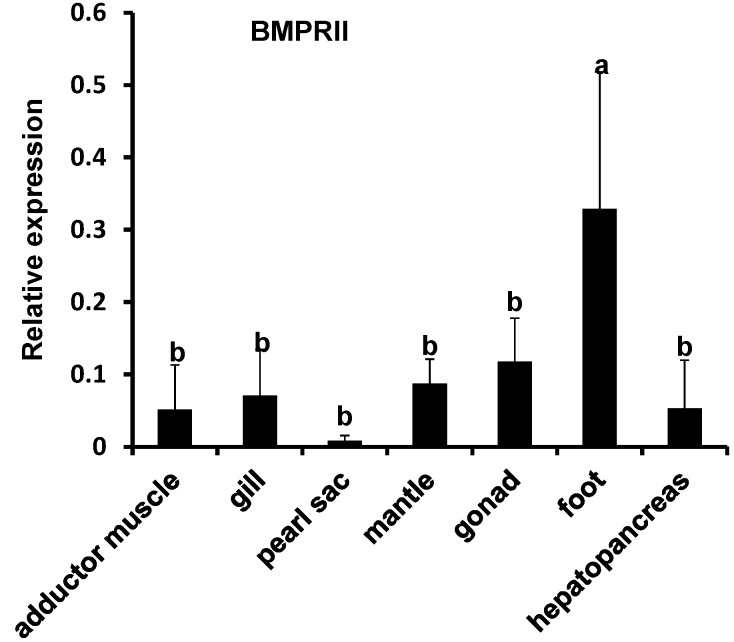
Expression pattern of Pm-BMP7 and its receptors mRNA in different tissues by qRT-PCR. Each bar is a mean of the different tissues (adductor muscle, gill, pearl sac, mantle, hepatopancreas, gonad and foot) from the five pearl oysters. The pearl oyster *β-actin* gene is used as the reference gene. The significant difference is indicated by different letters (*p* < 0.05). The error bars correspond to the mean + standard error (SE).

Studies have shown that BMP7 regulates cell proliferation and differentiation by signaling through two structurally related type I (BMPRI) and type II receptors (BMPRII). Two unigenes encoding Pm-BMPRI and Pm-BMPRII were obtained in the transcriptome database of *P. martensii.* The unigene sequences can be found in Figure S1. The qRT-PCR was performed to determine the tissue specific expression of Pm-BMP7 and its receptors in the adductor muscle, gill, pearl sac, mantle, hepatopancreas, gonad and foot with β-actin as the internal reference. The Pm-BMP7 mRNA was highly expressed in the mantle, which is shell formation related-tissue (Figure 4). Similarly to the Pm-BMP7 expression profile, Pm-BMPRI mRNA was highly expressed in the mantle, while Pm-BMPRII was highly expressed in the foot.

### 2.4. Functions of Pm-BMP7 in Shell Formation

**Figure 5:**
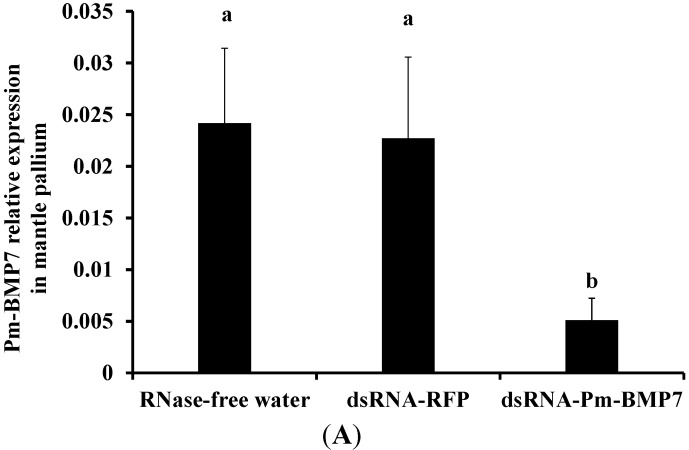

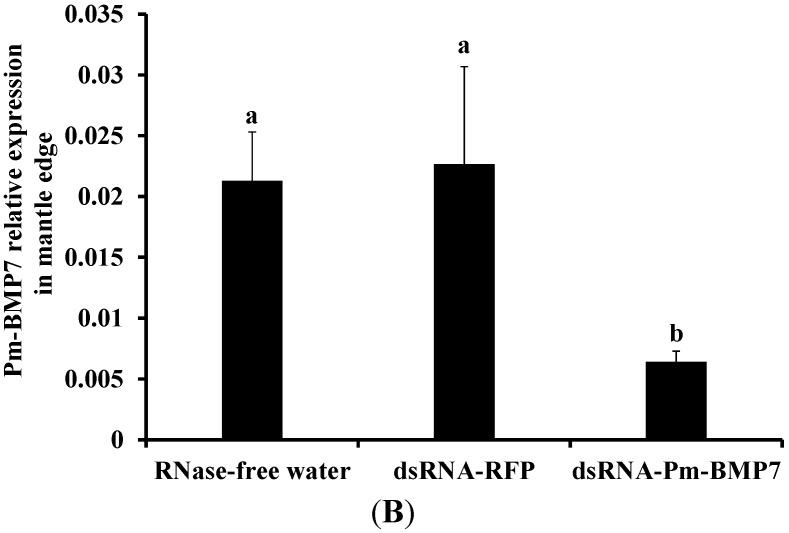
Relative expression of Pm-BMP7 mRNA in the mantle pallium (**A**) and the mantle edge (**B**) by RNAi. qRT-PCR was performed with RNA samples from the controls (RNase-free water, dsRNA-RFP) and the dsRNA-Pm-BMP7 group 8 days after the second injection, and five individuals are tested in each group. The pearl oyster *β-actin* gene is used as the reference gene. The significant difference is indicated by different letters (*p* < 0.05). The error bars correspond to the mean + SE.

To further investigate the function of *Pm-BMP7* gene on shell biomineralization *in vivo*, RNAi technology was applied to inhibit the expression of the *Pm-BMP7* gene. The controls were RNase-free water and dsRNA-RFP injected-groups. qRT-PCR was employed to measure the mRNA levels of Pm-BMP7 in the mantle pallium, which is involved in the nacre formation, and in the mantle edge, which is involved in the prismatic layer formation 8 days after the second injection. Compared with that from the control groups, the expression of the *Pm-BMP7* gene in the dsRNA-Pm-BMP7 injected group was down-regulated to approximately 22% in the mantle pallium (Figure 5A) and 30% in the mantle edge (Figure 5B).

**Figure 6:**
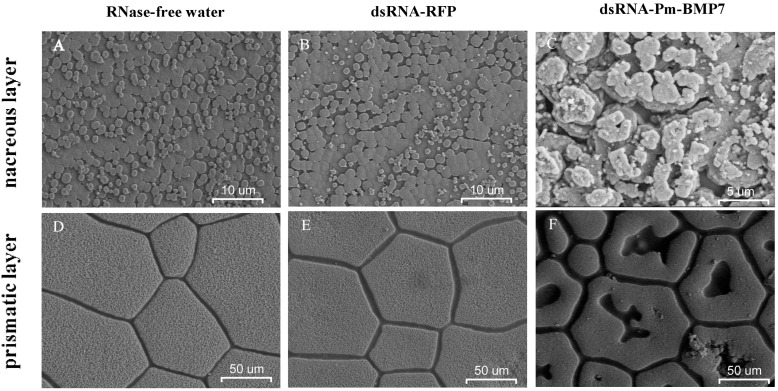
SEM images of the nacre (**A**–**C**) and the prismatic (**D**–**F**) layer after RNAi. The shells were derived from the pearl oysters injected with RNase-free water (**A**,**D**), dsRNA-RFP (**B**,**E**) and dsRNA-Pm-BMP7 (**C**,**F**). The bars are 10 μm in the nacre images and 50 μm in the prismatic layer images.

Then, the inner surface microstructure of the shells from each group was scanned using scanning electron microscope (SEM) 8 days after the second injection. The surfaces of the shells in the controls had the same normal well-defined type of microstructure. In the nacre, small hexagonal aragonite flat tablets were packed together to produce a stair-like growth pattern (Figure 6A,B), and in the prismatic layer, hexangular or pentangular column calcite crystals were tightly arranged (Figure 6D,E). However, in the dsRNA-Pm-BMP7 injected-group, aragonite tablets of the nacre presented a disordered growth status (Figure 6C), and calcite crystals of the prismatic layer displayed obvious holes (Figure 6F). These results suggested that Pm-BMP7 plays a potential role in the nacre and prismatic layer formation process of the shell.

## 3. Discussion

In this report, we cloned and identified pearl oyster Pm-BMP7 and found it to be highly similar in size and sequence to BMP7 from other species. All BMPs are synthesized as large precursors, containing a signal peptide, a pro-domain and a mature peptide [5]. This precursor protein is usually cleaved at an RXXR site in the pro-domain to release a mature carboxy-terminal segment of 110–140 aa. In vertebrates, every mature BMP shares seven conserved cysteines, six of which build a cystine knot by forming three intrachain disulfide bonds to resist heat, denaturants and extreme pH levels. The remaining cysteine residue forms an interchain disulfide bond that links two monomers into active hetero- or homo-dimers [21], whereas this unique cysteine was absent in the mature Pm-BMP7 protein. The same phenomenon took place in those from *C. gigas* and *T. granosa*. Our findings raise a possibility that BMP7 in the mollusks might not form dimers or might form noncovalently linked dimers between the subunits, which could be dynamic and subject to regulation.

The tissue expression patterns showed Pm-BMP7 distributed widely in all detected tissues of *P. martensii* with the highest expression in the mantle, which is the main shell formation related-tissue. Miyashita *et al.* [12] obtained the consistent result that the expression of BMP2 from *P. martensii* was predominant in the mantle by Northern blot and *in situ* hybridization, proposing BMP2 played a key role in the nacre formation. Iijima *et al.* [14] found BMP2/4 from *Lymnaea stagnalis* was expressed in the right-hand side ectoderm of the shell gland and in the invaginating stomodaeum, indicating BMP2/4 had a role in shell formation. Zoccola *et al.* [16] reported that BMP2/4 in coral was specifically synthesized by the calicoblastic epithelium, suggesting BMP2/4 played a role in coral skeletogenesis.

To further elucidate the role of the *Pm-BMP7* gene in shell formation, RNAi technology was used to inhibit the expression of Pm-BMP7. Compared with that from control groups, the expression of the *Pm-BMP7* gene was reduced by approximately 78% in the mantle pallium and 70% in the mantle edge. Together with the SEM images of the shells, we found a disordered growth status in the aragonite tablets and obvious holes in the calcite crystals in the dsRNA-Pm-BMP7 injected group. Therefore, these results suggest that Pm-BMP7 plays a crucial role in both the nacre and prismatic layer formation process of the shell.

BMPs are important factors with inducing activity in the process of bone formation, and can induce mesenchymal stem cells to differentiate into osteoblasts, so as to produce new bone tissue in vertebrates [22]. Studies have shown that BMP7 mediates its biological effects by binding and bringing together two different but related BMP receptors (BMPRI and BMPRII). During BMP7 mediated-signal transduction, BMP7 firstly binds to the BMPRII receptor, which recruits and transphosphorylates the BMPRI receptor [23]. Activation of BMPRII initiates the downstream pathway and activates specific target genes. Thus, formation of a heterodimeric complex between BMPRI and BMPRII receptors is required for BMP7 mediated-signal transduction. In mollusks, mantle epidermal cells secrete matrix proteins to control the CaCO_3_ polymorph (calcite, aragonite), the size, the shapes of the crystallites, and finally, the texture of the shell [2]. It is possible that Pm-BMP7 *in vivo* plays a key effect on the transformation and update of mantle epidermal cells. Over expression of Pm-BMP7 and Pm-BMPRI in the mantle indicated Pm-BMP7 might be able to induce the undifferentiated cells into epidermal cells and act as an active factor to regulate shell formation. The expression of Pm-BMP7 and its receptors were considerably low in the pearl sac, which was functional in pearl formation; this phenomenon is also mentioned by Shigeharu Kinoshita [24]. One possibility explained by Shigeharu Kinoshita is the different cellular environments between the mantle and pearl sac. The pearl sac is one very thin tissue. During shell formation, epithelial cells in the outer mantle secrete organic matrix and then calcification is carried out in the fluid-filled extrapallial space. Compared with the mantle, the extrapallial space in the pearl sac is supposed to be small. Therefore, the low expression of some genes may be sufficient for calcification in the pearl sac.

BMPs were first discovered in decalcified bone. Urist *et al.* [4] implanted decalcified bone extracts in the muscles of animals, found new bone deposition after 4–6 weeks, and later proved the active factor of inducing bone formation was BMPs. That is to say, BMPs were not only a bone-inductive factor, but also a major component of bone. Meanwhile, BMP7 was also discovered in the purified bone extract [25,26]. Interestingly, experiments *in vivo* and *in vitro* have shown that the nacre [27,28], especially its water-soluble matrix fraction [29,30], can induce pre-osteoblast differentiation, finally leading to bone formation. This phenomenon indicates that the nacre may contain one or more signal molecules for inducing osteogenesis. Hence, it was assumed that the inducing osteogenesis factor existing in both the nacre and the bone shared some homologous components, such as BMPs. Together with our results, we propose the lack of the Pm-BMP7 protein by RNAi may lead to its non-deposition on the shells, and finally result in the abnormal characteristics of the shell.

In conclusion, we have got the full length of *Pm-BMP7* cDNA from *P. martensii*, and analyzed the characteristic of its ORF and peptide sequence. Tissue expression analysis demonstrated *Pm-BMP7* and *Pm-BMPRI* were highly expressed in the mantle, while *Pm-BMPRII* was highly expressed in the foot. Using RNAi technology, we have identified Pm-BMP7 participated in shell formation. To better understand how Pm-BMP7 regulates these processes, further studies on the signaling pathway are required.

## 4. Experimental Section

### 4.1. Experimental Animals, RNA Extraction and cDNA Synthesis

Adult Pearl oysters (about 2 years of age) were obtained from Liushagang, Zhanjiang, Guangdong, China. The samples were cultured at 25–27 °C in tanks with recirculating seawater for 3–5 days before the experiment. Different tissues from the pearl oysters were acquired and immediately stored in liquid nitrogen until usage.

Total RNA was prepared using Trizol reagent (Invitrogen, Carlsbad, CA, USA) according to the manufacturer’s instructions. RNA quantity was evaluated by measuring OD_260_/OD_280_ with NanoDrop ND1000 spectrophotometer (Thermo scientific, Waltham, MA, USA), while RNA integrity was determined by fractionation on a 1.0% agarose gel and staining with ethidium bromide. Total RNA (1 μg) was used as the template for the RT-reaction with M-MLV reverse transcriptase (Promega, Madison, WI, USA) and random primer.

### 4.2. Rapid Amplification of cDNA Ends (RACE)

A partial sequence of the *BMP7* gene was obtained from the pearl sac transcriptome of *P. martensii*. Based on this fragment, the specific primers were designed. To increase specificity and sensitivity, nested-PCR was performed. The outer and inner primers were shown in Table 1.

**Table 1:** Primer list used in this study

Primer Name	Primer Sequence (From 5' to 3')	Application
BMP7-5' outer	TTAAGCTGCGATGAGCACTCGCTCACC	RACE
BMP7-5' inner	GAGCACTCGCTCACCCACGTTAAAGAT	RACE
BMP7-3' outer	TCCTCCATTAACGCCACCAATCACGC	RACE
BMP7-3' inner	TCAATAAATACGAGGCTCCAAGCCCC	RACE
BMP7-F	GCGCACTGCAATACGAAGA	qRT-PCR
BMP7-R	GGAGGACCCTAACGGAAAG	qRT-PCR
BMPRI-F	AAGGCAAAGATGGAGGAAC	qRT-PCR
BMPRI-R	TCTCGTGGAACTGGGTGAT	qRT-PCR
BMPRII-F	GAGACAAGTTTAAGCCTACCGT	qRT-PCR
BMPRII-R	GAATCCCAAGTCACCTATCACA	qRT-PCR
β-actin-F	GTGTAAGGCGGGGTTTGCT	qRT-PCR
β-actin-R	GGGTCCTTCAGCGTTAGTATCTT	qRT-PCR
dsRNA-BMP7-F	GCGTAATACGACTCACTATAGGGTCTAAAGAATCAGTGTTGGGGAGTG	RNAi
dsRNA-BMP7-R	GCGTAATACGACTCACTATAGGGGTTAATGGAGGACCCTAACGGAAAG	RNAi
dsRNA-RFP-F	GCGTAATACGACTCACTATAGGGGAGCTGGTTTAGTGAACCGTCAGA	RNAi
dsRNA-RFP-R	GCGTAATACGACTCACTATAGGGAAAACCTCTACAAATGTGGTATGGC	RNAi

The single-strand cDNA for all RACE reactions was prepared from the total RNA of the mantle using SMART RACE cDNA Amplification Kit (Takara, Dalian, China) according to the manufacturer’s instructions. For the first PCR, the synthesized cDNA was used as the template. For the second PCR, the first amplicated product was used as the template. The PCR program was proceeded as follows: 94 °C for 5 min, 30 cycles of 94 °C for 30 s, 94 °C for 30 s, 68 °C for 30 s and 72 °C for 1 min, and a final elongation step of 72 °C for 10 min.

### 4.3. DNA Sequencing and Sequence Analysis

The purified PCR products, containing the 5' and 3' ends, were subcloned into pMD-18T vector (TAKARA, Japan), transformed into DH-5α and sequenced. The full-length cDNA of *Pm-BMP7* gene was analyzed using the BLAST program (http://www.ncbi.nlm.nih.gov/). The open reading fragment (ORF) was estimated using ORF Finder (http://www.ncbi.nlm.nih.gov/gorf/orfig.cgi). The amino acid sequence was characterized with DNAMAN and analyzed with expasy program (http://prosite.expasy.org/). The signal peptide was predicted by SignalP-4.0 (http://www.cbs.dtu.dk/services/SignalP/). The mature protein molecular weight and theoretical pI were analyzed using program tool (http://web.expasy.org/cgibin/protparam/protparam). Multiple alignments were created using the ClustalX (Des Higgins and Paul Sharp, Dublin, Ireland) and BioEdit softwares (Bioedit Company, Manchester, Britain).

### 4.4. Quantitative Real-Time PCR (qRT-PCR) Assay

The sequences of the specific primers are shown in Table 1. The qRT-PCR assay was performed using Thermo Scientific DyNAmo Flash SYBR Green qPCR Kit (Thermo Scientific) according to the manufacturer’s instructions and was conducted through the Applied Biosystems 7500/7500 Fast Real-time system (Applied Biosystems, Foster City, CA, USA). β-actin was used as the reference gene to verify the successful reverse transcription and calibrate the cDNA template. In a 96-well plate, each sample was run in triplicate, along with the reference gene. The PCR program was conducted as follows: 4 min at 95 °C, 40 cycles (each cycle was 30 s at 95 °C, 15 s at 58 °C and 15 s at 72 °C).

### 4.5. RNA Interference (RNAi) Experiment

RNAi was performed to test the function of Pm-BMP7 on shell formation *in vivo*. Sequence specific primers (Table 1) were designed and used to amplify the specific sequences from the synthesized cDNA. The controls were: RFP (red fluorescent protein) gene, which is not expressed in *P. martensii* and RNase-free water. Ten individuals were used in each treatment.

The PCR products were purified using EasyPure Quick Gel Extraction Kit (TransGen, Beijing, China). The dsRNA was synthesized by the T7 RNA polymerase (Thermo Scientific), and the template DNA was digested by RNase free DNase I (Thermo). The integrity and quantity of the dsRNA were verified as previously described. The dsRNA-Pm-BMP7 was diluted to 80 μg/100 µL with RNase-free water, 100 µL solutions were injected into the muscle of *P. martensii* (2 years old, shell length-ranged between 5 and 6 cm) for the first time, and the same doses were injected 5 days after the first injection. For the control groups, the same volume of RNase-free water and 80 μg of dsRNA-RFP in RNase-free water was separately injected each time.

The total RNA was extracted from the mantle pallium and the mantle edge 8 days after the first injection and the first-strand cDNA was synthesized. The expression levels of the *Pm-BMP7* gene after RNAi was measured by qRT-PCR with β-actin as the internal reference. The corresponding shells were collected, washed with Milli-Q water, air-dried, and then cut into pieces (1 cm × 1 cm). The nacre and the prismatic layer of the shells of each group were observed by an FEI Quanta 200 SEM (FEI, Hillsboro, OR, USA).

### 4.6. Statistical Analysis

The data were analyzed using one-way ANOVA in SPSS 19.0 (IBM, Chicago, IL, USA). A *p*-value less than 0.05 (*p* < 0.05) was considered as statistical significance.

## 5. Conclusions

In summary, we obtained the full-length cDNA of Pm-BMP7, analyzed the features of its ORF and peptide sequence, and detected the expression pattern of different tissues in *P. martensii*. We further identified the function of Pm-BMP7 by RNAi technology. The results indicated that the obtained Pm-BMP7 in this study played a crucial role in the nacre and prismatic layer formation process of the shell. To better understand how Pm-BMP7is involved in these processes, further studies on the signaling pathways are required.

## Supplementary Materials

Supplementary figure can be found at http://www.mdpi.com/1422-0067/15/11/21215/s1.
